# Vertical Variation of Nonpoint Source Pollutants in the Three Gorges Reservoir Region

**DOI:** 10.1371/journal.pone.0071194

**Published:** 2013-08-12

**Authors:** Zhenyao Shen, Lei Chen, Qian Hong, Hui Xie, Jiali Qiu, Ruimin Liu

**Affiliations:** State Key Laboratory of Water Environment Simulation, School of Environment, Beijing Normal University, Beijing, P.R. China; Dowling College, United States of America

## Abstract

Nonpoint source (NPS) pollution is considered the main reason for water deterioration, but there has been no attempt to incorporate vertical variations of NPS pollution into watershed management, especially in mountainous areas. In this study, the vertical variations of pollutant yields were explored in the Three Gorges Reservoir Region (TGRR) and the relationships between topographic attributes and pollutant yields were established. Based on our results, the pollutant yields decreased significantly from low altitude to median altitude and leveled off rapidly from median altitude to high altitude, indicating logarithmic relationships between pollutant yields and altitudes. The pollutant yields peaked at an altitude of 200–500 m, where agricultural land and gentle slopes (0–8**°)** are concentrated. Unlike the horizontal distributions, these vertical variations were not always related to precipitation patterns but did vary obviously with land uses and slopes. This paper also indicates that altitude data and proportions of land use could be a reliable estimate of NPS yields at different altitudes, with significant implications for land use planning and watershed management.

## Introduction

After decades of working to reduce emissions from point sources, problems regarding nonpoint source (NPS) pollution have been highlighted, with agriculture being the largest contributor [1]. The three main forms of NPS pollutants are sediments, nutrients and pesticides [2], the effects of which are well documented [3]–[5]. Researchers have revealed that NPS pollution may come from a wide range of dispersed sources though a complex combination of physical, chemical and biological processes [6]. From an environmental point of view, there is a dire need to gain insights into the spatial variations of NPS pollution, which are essential for analyzing these complex problems in drainage basins.

In general, the spatial distributions of NPS pollution can be quantified by monitoring or modeling methods. In respect to monitoring strategy, detailed measured data are collected and the spatial variations of water quality can be analyzed by comparing those measured data. However, water quality degradation often results from multiple sources and separating the impacts by monitoring methods is very difficult and costly, especially for a large basin [7]. Watershed models can facilitate in identifying individual sources of NPS pollution and evaluating the decision schemes for watershed management. Up to now, many models have been developed for identifying the spatial distributions of NPS pollution [8]. Some of these models, such as Export Coefficient Model [9], are based on empirical equations and cannot always provide sufficient explanations for those complex watershed processes [10]. By contrast, those physically-based models can simulate the hydrologic and water quality responses at varying scopes and locations [11]. In addition, those physically-based models are usually coupled with the geographic information system (GIS) which can compile extensive input database and visualize the model results. Information of watershed characteristics can be extracted and analyzed with convenience of GIS techniques which are usually integrated into these watershed models. The most commonly-used watershed models are the Soil and Water Assessment Tool (SWAT) model [12], Agricultural Nonpoint Source pollution (AGNPS) model [13], Annualized Agricultural Nonpoint Source pollution (AnnAGNPS) model [14], and Hydrological Simulation Program - Fortran (HSPF) [15].

Currently, the GIS techniques have provided a reliable platform for integrating vertical and horizontal information within a basin. Within such a framework, altitude data are generally extracted from a Digital Elevation Model (DEM) [16], and this 3-dimensional information has been widely applied in studies on atmospheric pollution [17], [18]. However, there is current interest in integrating the GIS platform to project large volumes of meteorological and geophysical data into horizontal information to study NPS pollution [19], regardless of whether vertical variations occur. Indeed, altitude is the key attribute of topography and has a direct impact on physical parameters such as precipitation, solar radiation, temperature and soil chemistry [20], [21]. Altitude is also essential in other environmental factors, including slope length, slope degree and other properties [22], [23]. Researchers have reported that altitude has an impact on geomorphologic processes such as surface runoff, soil erosion and landslides in hilly regions [24], [25]. Land use changes, landscape dynamics and other human activities are therefore related to the terrain and altitude [26], especially in the mountainous areas. Therefore, those projected horizontal distributions of NPS pollution is a consequence but may not be the cause of NPS pollution because this information should make reference to specific vertical processes. As far as we know, there has been no attempt to incorporate vertical variations for the analysis of NPS pollution, which should draw increasing attention due to continued hilly urbanization, increased deforestation, and changed precipitation with global warming [27].

The objective of this paper is to contribute new insights into vertical variations to capture the complex features of NPS pollution. The study was performed in the Three Gorges Reservoir Region (TGRR) by: 1) exploring the spatial distributions of sediment, nitrogen (N), and phosphorus (P) using the Soil and Water Assessment Tool (SWAT); 2) establishing the relationships between land use, slope and altitude; and 3) characterizing the vertical variations of sediment, N and P yields in the TGRR.

## Materials and Methods

### Watershed Description

**Figure 1:**
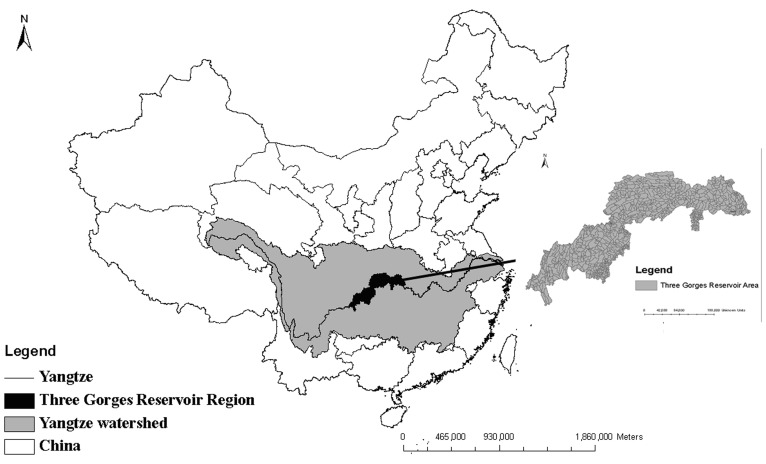
The location of the Three Gorges Reservoir Region.

The Three Gorges Reservoir, which is by far the world’s largest hydropower project, completed its first filling stage in 2003 and reached its maximum designed water level in 2008. Geographically, the TGRR, with a total area of approximately 58,000 km^2^, is located in the transitional zone from the Tibetan Plateau in the west to the east rolling hills and plains of China between latitudes 28°10′ and 32°13′N and longitudes 105°17′ and 110°11′E (Fig. 1). The topography is complex, with over 74% of the landscape being mountainous and 21.7% being low hills. The land uses include cropland (39%), grassland (13%) and forest (46%), while the main soils are purplish soils (48%), limestone earths (34%) and yellow (16%) earths. The average precipitation is approximately 1400 mm, 80% of which occurs from April to October. The highest and lowest annual temperature ranges from approximately 27°C to 29°C and 6°C to 8°C, respectively.

When water levels were driven up by the Three Gorges Reservoir, hilly reclamation and deforestation continued to increase above the 175-m inundation line [28], [29]. Due to the special geography and structure of the agriculture in the TGRR, the soil loss is serious and the eco-environment is vulnerable. Additionally, after the water was cut off, the water velocity was reduced and the retention time of pollutants prolonged. The water quality challenge has never been greater than now, as indicated by the soil erosion in the uplands and algal blooms in the aquatic environment [29].

### Model Description and Preparation

#### Model description

The ArcSWAT model, developed by Arnold et al. [12], was used to develop the necessary input files. The SWAT components include weather generation, hydrology, soil erosion, crop growth, nutrient leaching and agricultural management [30]. The hydrology calculation was based on the curve number method and the Green-Ampt infiltration method [31]. The sediment yield was estimated by the modified soil loss equation [32]. Runoff, sediments and nutrients were calculated for each Hydrologic Response Unit (HRU) and then routed in stream using the QUAL2E model [33]. More information about the SWAT model can be found in Douglas et al. [30] (Methods S1).

#### Data description

Taking into account the study needs and data availability, the digital layers for altitude, land use and soil were constructed. DEM data at a scale of 1∶25,000 published by the Institute of Geographical and Natural Resources Research, China, were used. Land-use data were interpreted from a 1∶100,000 Thematic Mapper image and the proportions of land uses were treated as constants during the simulation period. A soil map at a scale of 1∶1,000,000 and the related physical data were obtained from the Institute of Soil Science, Chinese Academy of Sciences. The daily precipitation, relative humidity, solar radiation, wind speed and air temperature data, measured by 49 weather stations from 1980 to 2010, were obtained from the State Meteorological Data Sharing Service System (http://cdc.cma.gov.cn). Crop information, including tillage, irrigation and the amount of fertilizer used, was based on statistical data from local bureaus as well as field investigations in several local watersheds. Rice, potato, sweet potato and corn were selected as the main crops due to the cultivated areas and the amount of fertilizer. However, no clear records of management practices were available. To compensate for this lack, the average fertilizer rates in each village were calculated for cultivated crops and all agricultural areas were assumed to be tile drained.

#### Model preparation

In this study, the TGRR was delineated into 613 sub-watersheds interconnected by a stream network, and each sub-watershed was divided further into HRUs by setting 0% thresholds of land use, soil type and slope to accurately capture even small areas. In our previous study [34], we introduced a small-scale watershed extended method (SWEM) for parameter calibration in the TGRR (Methods S2). The detailed processes involve: 1) model calibration- a process of generating model parameter groups for representing different parts of the TGRR (in terms of the watersheds of the Yulin, Xiaojiang, Daning and Xiangxi); 2) extended modeling- running the well-calibrated models in the corresponding parts of the TGRR (Figure S1). The measured flow and water quality data were obtained from the Changjiang Water Resources Commission and the parameter groups were generated by the SWAT-CUP [35]. The Nash-Sutcliffe efficiency coefficient (*E_NS_*) [36] was used to quantify the degree of fit between the simulated data and the measured data.
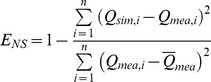
(1)Where, 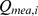
is the ith observation for the constituent being evaluated, 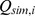
is the predicted value for the constituent being evaluated, 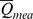
is the mean value of observed data for the constituent being evaluated, and n is the total number of observations.

The values of *E_ns_* in the respective sub-watersheds ranged from 0.53 to 0.94 for the stream flow, 0.53–0.94 for sediment, 0.60–0.84 for total P (TP), 0.47–0.80 for nitrate-N and 0.41–0.81 for NH_4_-N. The detailed processes of the model calibration and validation can be found in our previous study [34], [37]. With the groups of calibrated parameters, the extended simulation was conducted by running the well-calibrated SWAT models in the entire TGRR.

#### Data analysis

Following calibration, a 10-year (2000–2009) simulation was performed to isolate variability of climate, land use, crop rotations and runoff regime which may mask the effect of vertical variation [38]. The DEM and land use maps were divided into two matrixes of 4500 rows by 5500 columns, with a cell size of 100*100 m. A mask layer was applied to all cells to avoid noise in the statistical processing of the data, and 6,200,000 cells remained after this selection. Topographic data such as altitude, slope and land use were generated for each cell, and the flow, sediment and nutrient yield were calculated from SWAT outputs. The total yields were defined as the summary of corresponding cells.

## Results and Discussion

### The Vertical Variations of Land Use and Slope

**Figure 2:**
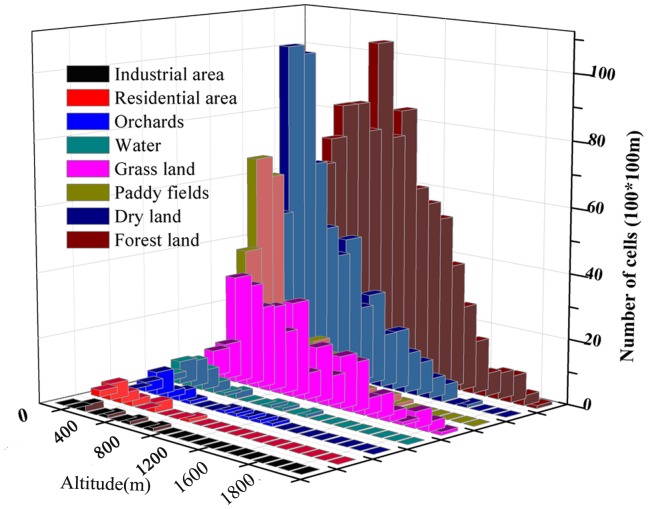
The vertical variations of land use.

**Figure 3:**
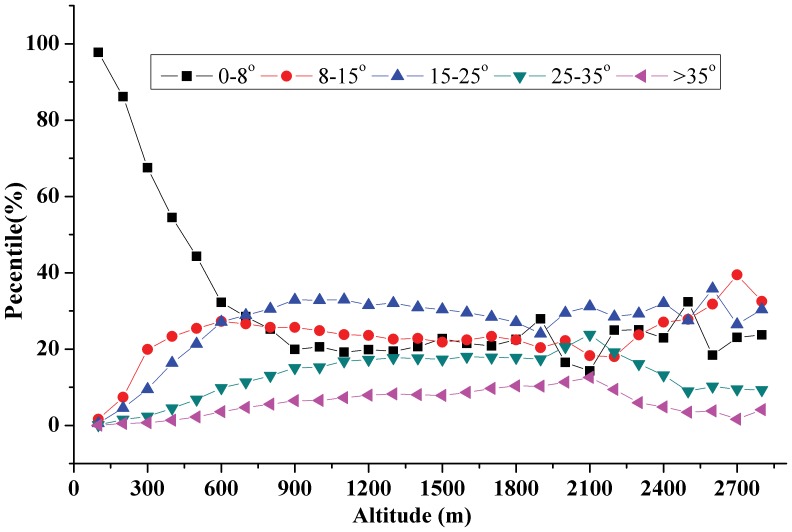
The vertical variations of slope.

Fig. 2 illustrates the vertical variations of the land uses at different altitudes. According to Fig. 2, the landscape area increases significantly from 0 m to 400 m and levels off rapidly from 400 m to 1600 m, while only slight declines can be observed when the altitude varies from 1600 m to 2100 m. Specifically, the land from 200 m to 1000 m was dominant in the TGRR, covering more than 71% of the entire area. The landscape area accounted for only 2% from 0 m to 200 m, 13% from 1000 m to 1500 m and 2% from 1500 m to 2100 m of the total area. Among the different land uses, agriculture (paddy field and dry land), forest and grassland were dominant in all altitudes but the vertical variations of these land uses were different. As illustrated in Fig. 2, the proportions of agriculture show obvious declines as the altitude increases, while those of forest and grassland show increasing trends. In particular, agricultural areas were concentrated among the altitudes between 200 m and 800 m. This vertical variation could be explained by most low-altitude areas below the 175-m inundation line having been submerged when the water levels were driven up by the Three Gorges Dam [29], [38]. These vertical variations of land uses were also indicated by other studies showing that farmers resettled in the low hilly areas and 80% of the arable farmland is distributed in the low hilly areas or valley terraces [39], [40]. In this study, the slope degrees were categorized into 0–8**°**, 8–15**°**, 15–25**°**, 25–35**°** and 35∼90**°**. The vertical variations of these slope degrees are analyzed in Fig. 3. In the TGRR, gentle slope (0–8**°)** made up the largest proportion (31%) of the entire area, while the land on median slope (8–25**°**) and steep slope (25–90**°**) accounted for 50% and 19%, respectively.

Fig. 3 also illustrates that the vertical variations of slope degree show obvious trends. Below the altitude of 800 m, the proportions of gentle slope gradually decreased as altitude increased, while the proportions of median and steep slopes increased slightly. Between the altitudes of 800 m and 2000 m, the proportions of gentle slope and median slope remained stable, while the proportions of steep slope continued to increase. Above the altitude of 2000 m, the landscape area was again dominated by gentle and median slopes. This result can be explained by the widely held view that the rock strength at high altitudes is normally high and that such altitudes usually have weathered rocks or rocks whose shear strength is much higher [25], indicating the high altitude areas in the TGRR are generally flat and have a convergent terrain.

### The Vertical Variations of NPS Pollution

**Figure 4:**
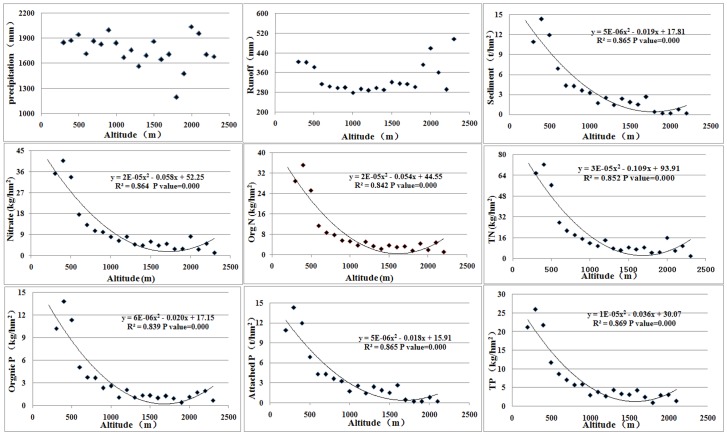
The vertical variations of precipitation and pollution yield.

Fig. 4 further characterizes the vertical variation of precipitation, sediment, N and P yields. Fig. 4 illustrates that precipitation did not vary significantly with altitude. This result is inconsistent with previous studies [16], [41] that demonstrated that precipitation increased with altitude due to the orographic effect, which lifted the air vertically and the condensation occurred due to adiabatic cooling. There are two probable reasons for this inconsistent variation. First, the climate in the TGRR is subtropical, with the annual mean temperature being 17°C, so adequate illumination may compensate for the orographic effect in the mountainous terrain. Second, a 10-year period was considered in the current study to represent the climatic variations. As rainfall is irregular in occurrence, duration and magnitude, this long period is equally true for a flattening effect of precipitation [41]. This paper indicates that NPS pollution did vary with altitude, even in the absence of different precipitation patterns related to altitude.

As illustrated in Fig. 4, the load intensities of all pollutants showed obvious declines from low altitude to high altitude. All variables peaked at the low altitude (200–500 m), where frequency of human actives is the highest. Specifically, soil erosion (above 500 t/(km^2^ a)) occurred in over 90% of the altitudes of the TGRR, while 33% of the areas were heavily eroded below the altitude of 500 m, with an erosion coefficient greater than 4,000 t/(km^2^ a). This result can be explained by the rock strength and vegetation cohesion making the high altitudes pollutant sinks, while the low altitude areas were normally prone to environmental vulnerability due to human disturbances [24], [25]. The logarithmic lines were generated to demonstrate the correlation between the pollutant yields and altitude (Fig. 4). As shown in Fig. 4, the regression results are significant, with the regression correlations being larger than 0.74.

**Figure 5:**
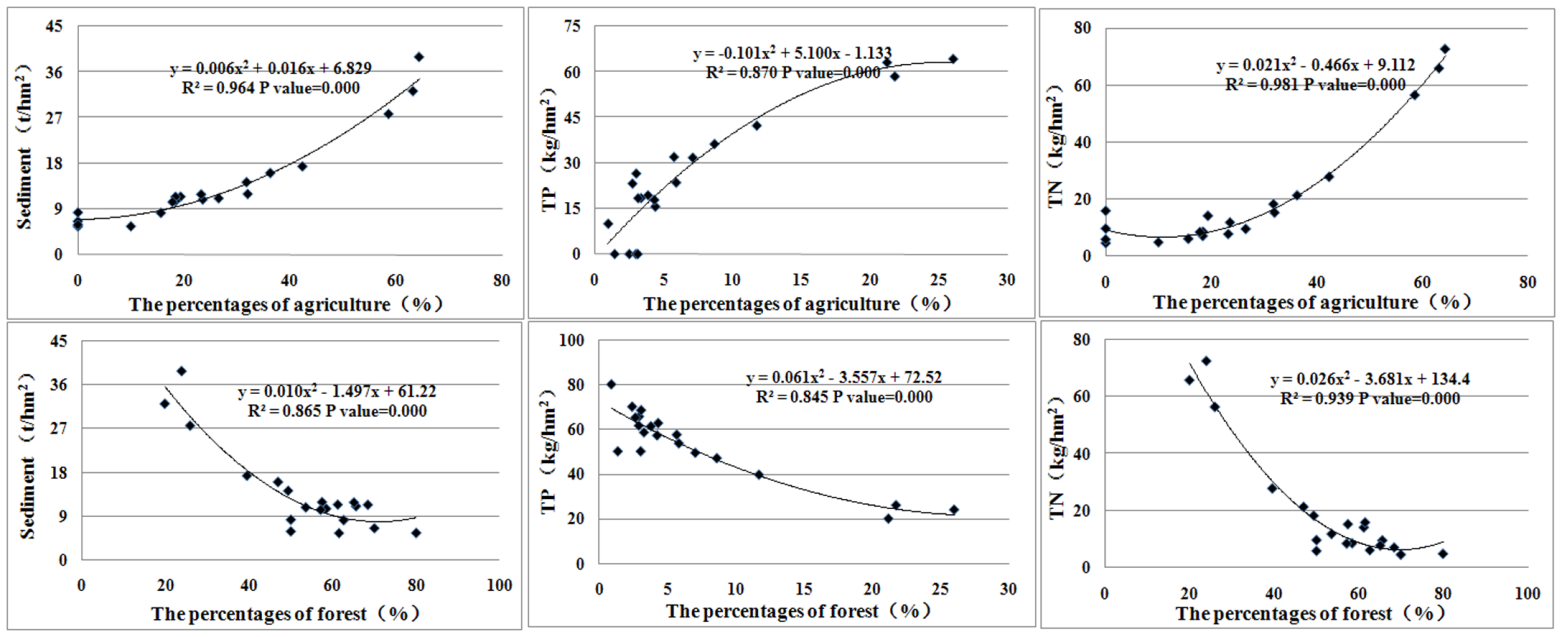
The relationship between pollutant yield and land uses in different altitudes.

The relationships among land use, slope and NPS yields were also explored. As illustrated in Fig. 5, the proportion of agricultural area was positively correlated with pollution yields, while that of forest was negatively correlated. For every 1% reduction in forest area, the load intensity increased by 0.01∼11.34 t/km^2^ for sediment, 0.15∼2.83 kg/km^2^ for TP and 0.40∼14.00 kg/km^2^ for total nitrogen (TN). The main reason for this result is that forest plants generally have a higher capability to hold and fix soil, while agricultural soil is either regularly over-fertilized or highly vulnerable to erosion [42]. In the TGRR, the agricultural area shrank at a high rate due to the Three Georges Reservoir, and there was no alternative but to rely on greater applications of fertilizer to ensure high productivity for the huge and growing population [29], [38], [43]. Specifically, the sediment yield increased slightly when the proportions of agriculture changed from 0% to 40%, and it showed a jump when the agriculture varied from 40% to 60%. This phenomenon could also be observed in P and N yields, from which the jumping points were obtained at proportions of 10% and 40%, respectively. For forest, the load intensity of sediment, TN and TP remained stable outside a relevant domain of 40%, 10% and 40%, respectively, and any change inside this proportion domain would have a greater impact on NPS yields. This phenomenon may be explained by the spatial distributions of the converted landscape pattern, which may mitigate certain discharges and may not always intensify the NPS pollution [44].

As shown in Table 1, the load intensity of sediment, organic N and attached P progressively rises with relief up to the median slope. The pollutant yield then declines toward steep slope. The most severe pollution risks typically occurred on the slopes between 8**°** and 15**°**, which is inconsistent with the widely held view that the downhill force is highest on steeper slopes [25]. However, the TGRR is major region for national environmental protection, and many projects, such as ‘Grain for Green Project’, have been implemented in steep slope areas to strengthen soil and reduce NPS yields. Additionally, human practices, including rotation, irrigation and tillage, have been conducted at the gentle slope and low altitude areas; therefore, gentler slopes, particularly in deeply human-impacted slopes, may increase hydrological connectivity and nutrient leaking, resulting in greater efficiency of delivery of P and N to surface waters [42].

**Table 1:** The load intensities of pollutants at different altitudes.

Slope	Sediment	Nitrate	Orgnic N	Attached P	Solute P	TN	TP
	t/hm^2^	kg/hm^2^	kg/hm^2^	kg/hm^2^	kg/hm^2^	kg/hm^2^	kg/hm^2^
0–8°	11.9	28.7	21.0	1.5	1.1	49.8	4.1
8–15°	23.0	17.0	23.4	2.0	0.6	40.5	4.1
15–25°	18.3	10.7	12.4	1.0	0.4	23.1	2.1
25–35°	15.8	5.7	6.1	0.7	0.2	11.8	1.3
>35°	16.2	3.8	3.7	0.5	0.2	7.5	0.9

### Conclusions

In this paper, the vertical variations of land use, slope and NPS yields were estimated and used for studying the behavior of pollutants in the TGRR. Based on our results, the NPS pollution showed an obvious decline from low to high altitude, with all variables peaking at the low altitude (200–500 m), where the frequency of human actives was the highest. The watershed manager can gain insight into vertical dynamics to develop site-specific policies using this spatial information. This paper indicates that the vertical variations of NPS pollution were not related to precipitation patterns but did vary with vertical variations of land uses and slopes. Therefore, altitude data and proportions of land uses can be regarded as a reliable estimate of NPS load intensity, especially in the mountainous areas. However, uncertainty of modeling outcomes must be estimated to establish the reliability of the simulated outputs. In the future, more detailed data should be used and more pollutants, such as pesticide, heavy metal and Polychlorinated Biphenyl, should be incorporated into the list of analysts.

## Supporting Information

Figure S1 **The overall framework for Small-scale watershed extended method (SWEM).**
(TIF)

Methods S1 **The description of Soil and Water Assessment Tool (SWAT).**
(DOC)

Methods S2 **The description of Small-scale watershed extended method (SWEM).**
(DOC)
