# Supplementary figures and images for: Vertical Variation of Nonpoint Source Pollutants in the Three Gorges Reservoir Region
Source: PLoS One. 2013 Aug 12;8(8):e71194. doi: 10.1371/journal.pone.0071194 (PMC3741353; doi:10.1371/journal.pone.0071194)

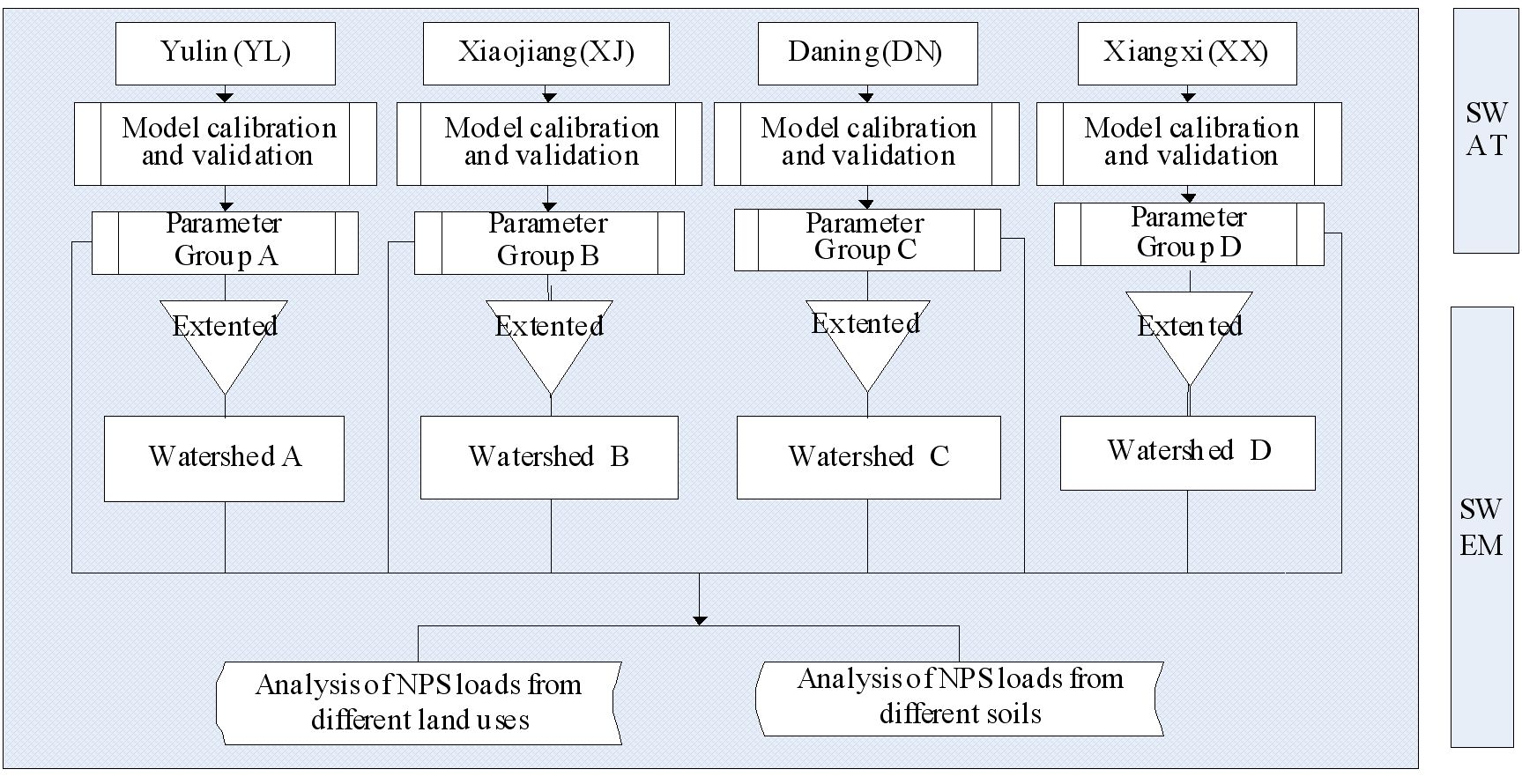

Supplement: Figure S1 — The overall framework for Small-scale watershed extended method (SWEM). (TIF) [file pone.0071194.s001.tif]
