# Supplementary material for: Vertical Variation of Nonpoint Source Pollutants in the Three Gorges Reservoir Region
Source: PLoS One. 2013 Aug 12;8(8):e71194. doi: 10.1371/journal.pone.0071194 (PMC3741353; doi:10.1371/journal.pone.0071194)
Supplement: Methods S1 — The description of Soil and Water Assessment Tool (SWAT). (DOC) [file pone.0071194.s002.doc]

**Methods S1: The description of Soil and Water Assessment Tool (SWAT)**

**Hydrological component**

The main driving force behind SWAT is the hydrological component. Hydrological processes are divided into two phases: (1) the land phase, which controls the amount of water, sediment, and nutrient loading in receiving waters; and (2) the water routing phase, which simulates movement through the channel network. For estimating surface runoff, daily rainfall data are chosen for the curve number (CN) method [1] and sub-daily data are chosen for the Green-Ampt infiltration method [2]. The SCS curve number equation is:

(S1)

Where *Q_surf_* is the accumulated runoff or rainfall excess (mm H_2_O); *R_day_* is the rainfall depth for the day (mm H_2_O); *I_a_* is the initial abstractions, which includes surface storage, interception, and infiltration prior to runoff (mm H_2_O); and *S* is the retention parameter (mm H_2_O).

**Sediment component**

The Modified Universal Soil Loss Equation (MUSLE) is used to estimate sediment yield at Hydrologic Research Unit (HRUs) level [3].

$Q_{sed}=11.8(Q_{surf}\cdot q_{peak}\cdot A_{hru})^{0.56}\cdot K_{usle}\cdot C_{usle}\cdot P_{usle}\cdot L_{usle}\cdot F_{CFRG}$ (S2)

where *Q_sed_* is the sediment yield on a given day (metric tons); *Q_surf_* is the surface runoff volume (mm H_2_O/ha); *q_peak_* is the peak runoff rate (m^3^/s); *A_hru_* is the area of the HRU (Hydrological response units) (ha); *K_usle_*, *C_usle_*, *P_usle_* and *L_usle_* is the USLE soil erodibility factor, cover and management factor, topographic factor; and coarse fragment factor, respectively.

**Nutrient component**

SWAT considers both anthropogenic contributions (manures, fertilizers, and point sources) and natural sources (organic matter, atmospheric deposition, and N-fixation) as nutrient inputs. The organic and mineral N and P fractions are simulated depending on the transformation and/or the additions/losses occurring within each pool [4]. A mass balance is calculated on a daily time scale to capture the series of changes addressed through equations for the respective processes. A single growth model in SWAT is used for simulating all crops based on the simplification of the EPIC crop model. The mass production and transport processes of SWAT are calculated at the HRU level (equation S3~S5) and combined at the outlet of each sub-watershed. Then the nutrients were routed through the channels, ponds, reservoirs, and wetlands to the watershed outlet, using the QUAL2E model [5].

$P_{surf}=0.001\times C_{orgP}\cdot\frac{Q_{sed}}{A_{hru}}\cdot\varepsilon_{P:sed}$ (S3)

$N_{surf}=0.001\times C_{orgN}\cdot\frac{Q_{sed}}{A_{hru}}\cdot\varepsilon_{N:sed}$ (S4)

$NO3_{surf}=\beta_{NO3}\cdot C_{NO3,mobile}\cdot Q_{surf}$ (S5)

Where *P_surf_**, N_surf_* and *NO3_surf_* is the amount of organic P, organic N and nitrate in surface runoff (kg /ha); *C_orgP_* , *C_orgN_* and *C_NO3,mobile_* is the concentration of organic P, organic N (g P/ metric ton soil) and nitrate (kg N/mm H_2_O) in the top 10 mm of soil; *ε_P:sed_* and *ε_N:sed_* is the P and N enrichment ratio; *β_NO3_* is the nitrate percolation coefficient.

**Reference**

1. USDA-SCS, Hydrology Sect. 4, Soil Conservation Service National Engineering Handbook; Washington, DC., 1972.
2. Williams, J. R., Flood routing with variable travel time or variable storage coefficients. *T. ASABE* **1976,** *12*, 100-103.
3. Arnold, J. G.; Srinivasan, R.; Muttiah, R. S.; Williams, J. R., Large area hydrologic modeling and assessment - Part 1: Model development. *J. Am. Water Resour. As.* **1998,** *34*, (1), 73-89.
4. Somura, H.; Takeda, I.; Arnold, J. G.; Mori, Y.; Jeong, J.; Kannan, N.; Hoffman, D., Impact of suspended sediment and nutrient loading from land uses against water quality in the Hii River basin, Japan. *J. Hydrol.* **2012,** *450*, 25-35; DOI: 10.1016/j.jhydrol.2012.05.032.
5. Brown, L. C.; Barnwell, T. O. The Enhanced Stream Water Quality Models QUAL2E and QUAL2E-UNCAS: Documentation and User Manual; Athens, 1987.
