# Supplementary material for: Vertical Variation of Nonpoint Source Pollutants in the Three Gorges Reservoir Region
Source: PLoS One. 2013 Aug 12;8(8):e71194. doi: 10.1371/journal.pone.0071194 (PMC3741353; doi:10.1371/journal.pone.0071194)
Supplement: Methods S2 — The description of Small-scale watershed extended method (SWEM). (DOC) [file pone.0071194.s003.doc]

**Methods S2: The description of Small-scale watershed extended method (SWEM)**

To compensate for data shortage, we introduced a SWEM in part of the TGRR, and further extended this SWEM to the entire TGRR [1]. The basic idea of the SWEM is that the parameter groups of several typical catchments among a large watershed can be extended to the surrounding areas until the NPS pollution loads in the whole watershed can be obtained. The detailed processes involve: 1) model calibration- a process of estimating model parameter groups in the watersheds of the Yulin, Xiaojiang, Daning and Xiangxi to generate parameter groups for representing different parts of the TGRR [1-3]; 2) extended modeling- running the well-calibrated models in the corresponding parts of the TGRR. The Sequential Uncertainty Fitting version 2, which is incorporated into SWAT-CUP [4], was adopted for parameter calibration and validation. The Nash-Sutcliffe efficiency coefficient (*E_NS_*) was used to quantify the degree of fit between the measured data and the simulated data.

$E_{NS}=1-\frac{\sum_{i=1}^{n} \left(Q_{sim,i}-Q_{mea,i}\right)^{2}}{\sum_{i=1}^{n} \left(Q_{mea,i}-\overline{Q}_{mea}\right)^{2}}$ (1)

Where, $Q_{mea,i}$is the ith observation for the constituent being evaluated, $Q_{sim,i}$is the predicted value for the constituent being evaluated, $\overline{Q}_{mea}$is the mean value of observed data for the constituent being evaluated, and n is the total number of observations.

**Reference**

1. Hong, Q.; Sun, Z.; Chen, L.; Liu, R.; Shen, Z., Small-scale watershed extended method for non-point source pollution estimation in part of the Three Gorges Reservoir Region. *Int. J. Environ. Sci. Tech.* **2012,** *9*, (4), 595-604; DOI:10.1007/s13762-012-0094-y.
2. Shen, Z.; Chen, L.; Liao, Q.; Liu, R.; Hong, Q., Impact of spatial rainfall variability on hydrology and nonpoint source pollution modeling. *J. Hydrol.* **2012,** *472-473*, 205-215; DOI:10.1016/j.jhydrol.2012.09.019.
3. Ouyang, W.; Hao, F. H.; Wang, X. L.; Cheng, H. G., Nonpoint source pollution responses simulation for conversion cropland to forest in mountains by SWAT in China. *Environ. Manage.* **2008,** *41*, (1), 79-89.
4. Abbaspour, K. C. SWAT-CUP2: SWAT calibration and uncertainty programs - a user manual; Department of Systems Analysis, Integrated Assessment and Modelling (SIAM), Eawag, Swiss Federal Institute of Aquatic Science and Technology: Duebendorf, 2008.
